# Practical Chemistry for Students and Physicians—Chapter First

**Published:** 1883-07

**Authors:** 


### Editorial.

---

### PRACTICAL CHEMISTRY FOR STUDENTS AND PHYSICIANS, CHAPTER FIRST.

(Continued from June Register, Page 572).

In the language of chemistry a molecule is the smallest particle into which any homogeneous body can be divided without losing its identity, as a molecule of water containing two atoms of hydrogen and one of oxygen.

Any further division, it is plain, would separate not molecules, not particles of water, but atoms. In the same chemical language, an atom is the minutest part of matter that can enter into chemical combination, that is, that can take part in forming a chemical compound, as hydrogen and oxygen uniting to form a molecule of water.

Weight, Quality and Quantity in respect to Atoms. These ultimate particles of matter differ among themselves in three particulars, viz: in weight, in the quality of their combining power and in the quantity of their combining power.

*Atomic Weight.*—The relative weight of any atom referred to hydrogen as 1, is its atomic weight. It is the least part by weight of any element, which can enter into chemical combination.

The old chemistry had, of course, its combining proportions, like the new, but it knew nothing of atomic weights. 1 and 8 were its combining proportions for the constituents of water, but 8 is not the atomic weight of oxygen; that is 16, yet 2 and 16, the proportions of the new chemistry, are precisely the same, in that respect, as 1 and 8.

*Positive Atoms.—Negative Atoms.*—Atoms are divided, in respect to their quality of combining power, into two classes, viz: positive atoms and negative atoms.

This classification is in virtue of an electro-chemical relation, subsisting among the elements themselves, but it is merely relative and not absolute. Chemical attraction is not electricity, though a few great chemists once thought it was.

This principle of combining power among the chemical elements, is the controlling fact in what is called the new nomenclature; it is the ground of distinction between *acids and bases*, and gives shape to the written formula and name of every chemical compound. Examples: When a solution of common salt is subjected to the influence of electricity, it is decomposed into chlorine and the metal sodium.

In the separation that takes place, the chlorine is seen to collect at the positive electrode of the battery, while the sodium atoms gather around the negative.

Hence the chlorine is negative in its relations to sodium, while sodium is equally positive in its relation to chlorine.

Another principle, still more important appears to depend upon this electro-chemical relation among the elements. When water combines with this negative chlorine it forms what is called a hydrate of chlorine, and is always in its nature an acid.

When the same water unites chemically with the positive sodium, on the other hand, it forms a hydrate again, but this hydrate is as strong a base as the other is an acid.

What is true in this respect of common salt, is true of nearly every other salt; it is composed of an acid negative radical, and of a basic positive root.

This is one of nature's mysterious laws, and can no more be understood in the present state of chemical knowledge than the nature of electricity.

If what is here stated of positive and negative atoms or radicals be remembered when the subject of notation and nomenclature is presented it will appear simple enough.

*Quantivalence.*—The quantity of combining power of an atom is the number of hydrogen atoms, which any element can combine with or be exchanged for. That is, if the combining power of the atom of hydrogen be taken as 1, that of all other atoms will be respectively 1, 2, 3, 4, 5, 6, or 7.

Hence the quantivalence of an element may be defined; the quantity of its combining power expressed in hydrogen units. It expresses the number of hydrogen atoms it can combine with or be exchanged for.*

Examples: An atom of chlorine, bromine, iodine or silver may be exchanged for one atom of hydrogen, and hence, these with all similar elements, have equivalent quantivalence. They are named from the Greek *monads.*

An atom of zinc, copper, oxygen and magnesium, can displace two atoms of hydrogen each. These elements, then, and all like them, have bivolent quantivalence, and are called from the Greek, *dyads*

An atom of nitrogen, antimony, arsenic or bismuth may be substituted for three atoms of hydrogen. These are of trivalent quantivalence, or Greek *triads.*

---

*Barker.

An atom of carbon, silicon, tin or platinum may be displaced by four of hydrogen. Quadrivalent quantivalence is, therefore, the measure of their combining power as regards quantity, and they are known from the Greek as *tetrads*.

In this way, all the elements may be grouped from one to the seventh degree of combining power with hydrogen.

Quantivalence was not even dreamed of by the old chemists; neither was the distinction of bases and acids on the ground of the electro-positive and negative relation of the elements.

*Variable Quantivalence.*—Although clearly established as a great chemical law, quantivalence is, nevertheless, not invariable.

Every student of chemisty knows that oxygen gas combines in several different proportions with both chlorine and nitrogen, forming as many very different chemical compounds.

A more familiar example to the student is that of chlorine and the metal mercury. Calomel, one of their compounds, contains only half as much chlorine as corrosive sublimate, another, the quantity of the metal being the same in both.

This variableness of the law of quantivalence has not yet been satisfactorily explained. There is quite a compensation for it, however, in the fact that it proceeds in every instance by a fixed law of its own—there is a method in its fickleness.

*The Law of Quantivalent Variation.*—The variableness of quantivalence *increases or diminishes always by* 2.

Consequently there are two arithmetical series possible in the expression of quantivalence; viz: 1 plus 2 equal to 3, 3 plus 2 equal to 5, 5 plus 2 equal to 7, the first series. The second is 2 plus 2 equal to 4, 4 plus 2 equal to 6. The first is composed of odd numbers and the second of even.

*Artiads and Parissads.*—These are Greek words that mean nothing more than what is already expressed by the words even and odd, but being technical terms in the new chemistry it is expedient to notice them.

1, 3, 5 and 7 are each perissad quantivalences; 2, 4 and

6, artiad. Examples: Iron in green vitriol or copperas is a dyad, in pyrite, a tetrad and in ferric trioxide a hexad. Iron, therefore, combines with other elements in the artiad series of 2, 4, 6.

Chlorine in hyperchlorous acid, combines with oxygen as a monad, in chlorous acid as a triad, in chloric acid as a pentad, and in perchloric acid, as a heptad. Hence the variable quantivalence of chlorine is expressed in the perissad series, 1, 3, 5, 7.

*The Two Series not Mutually Convertible.*—It is obvious from the nature of the law of variation of quantivalence that an artiad cannot be converted into a perissad, and vice versa.

Example: Let two be added to 1, the sum is 3, the next higher perissad. Add 2 again to 2 in the second series, the sum is 4, the next higher artiod of that; etc. for each figure in either series.

This is the whole of the theory of chemical quantivalence. And under the head of chemical notation and nomenclature, its use and applications will be found so simple and rational as to commend to every mind a subject, before regarded as only intricate and absurd.

*Chemistry.*—A comprehensive definition may now be given of Chemistry: "Chemistry is that branch of physical science which treats of the atomic composition of bodies and of those changes in matter which result from an alteration in the kind, the number or the relative position of the atoms which compose the molecule.*

